# Recent Advances in Nanozyme Sensors Based on Metal–Organic Frameworks and Covalent–Organic Frameworks

**DOI:** 10.3390/bios14110520

**Published:** 2024-10-23

**Authors:** Xingliang Cheng, Shuojiang Liu, Yuling Hu

**Affiliations:** School of Chemistry, Sun Yat-sen University, Guangzhou 510060, China; chengxliang@mail2.sysu.edu.cn (X.C.); liushj65@mail2.sysu.edu.cn (S.L.)

**Keywords:** metal–organic frameworks, covalent–organic frameworks, nanozymes, sensors

## Abstract

Nanozymes are nanomaterials that exhibit enzyme-like catalytic activity, which have drawn increasing attention on account of their unique superiorities including very high robustness, low cost, and ease of modification. Metal–organic frameworks (MOFs) and covalent–organic frameworks (COFs) have emerged as promising candidates for nanozymes due to their abundant catalytic activity centers, inherent porosity, and tunable chemical functionalities. In this review, we first compare the enzyme-mimicking activity centers and catalytic mechanisms between MOF and COF nanozymes, and then summarize the recent research on designing and modifying MOF and COF nanozymes with inherent catalytic activity. Moreover, typical examples of sensing applications based on these nanozymes are presented, as well as the translation of enzyme catalytic activity into a visible signal response. At last, a discussion of current challenges is presented, followed by some future prospects to provide guidance for designing nanozyme sensors based on MOFs and COFs for practical applications.

## 1. Introduction

Natural enzymes serve a crucial role as highly efficient and selective biocatalysts in various biochemical processes within organisms. However, their catalytic, sensing, and medical applications are limited due to issues like poor stability, high costs, challenging purification, and difficulties in large-scale production [1,2]. As a result, there is a growing interest in finding effective alternatives to natural enzymes [3,4,5,6,7]. Nanozymes, which exhibit enzyme-like properties, have emerged as promising substitutes. They demonstrate catalytic activity comparable to natural enzymes while offering advantages such as increased stability, ease of synthesis, modifiability, and lower costs [8,9,10,11,12,13].

In recent years, metal–organic framework (MOFs) and covalent–organic frameworks (COFs) have become the most concerned platforms for the design and modification of nanozymes among varieties of nanomaterials [14,15,16,17]. MOFs and COFs, consisting of metal ions and organic ligands connected by coordinate bonds, or organic nodes connected by covalent bonds, provide advantages like a high surface area, a tunable pore size, and easily modifiable functional sites. They have found widespread applications in biosensing, separation, drug delivery, etc. [18,19,20]. Numerous MOFs and COFs have been discovered to possess enzyme-like activity, often exhibiting higher catalytic activity, selectivity, reusability, and tunable catalytic performance compared to traditional nanozymes [21,22,23]. Additionally, MOF and COF nanozymes can easily be combined with other nanomaterials or biomolecules such as metal nanoparticles, DNA, proteins, and cells to create multifunctional hybrid materials with unique properties [24,25,26,27].

**Scheme 1:**
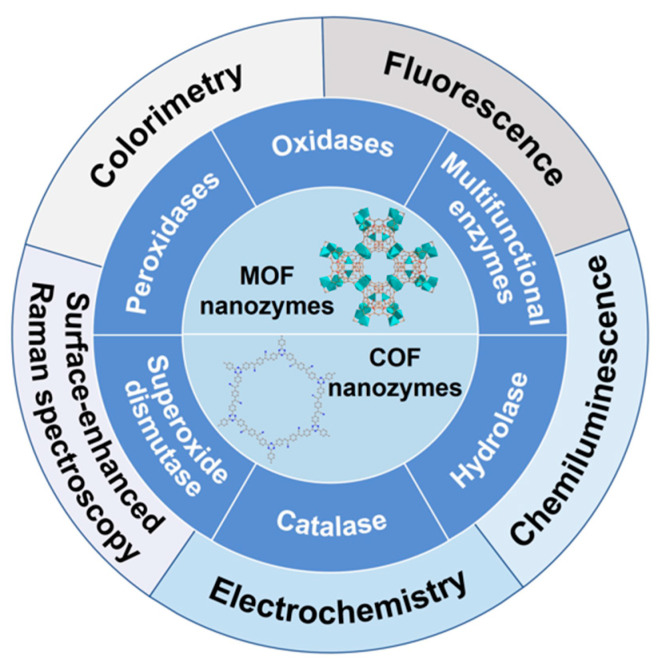
Summary of sensing analysis based on MOF and COF nanozymes.

To date, limited reviews have been reported on intrinsically enzyme-active MOF or COF nanozymes and their applications in therapeutics or detection, most of which are based on MOFs loaded with enzymes or combining MOFs with catalysts [18,19,20,28]. In this review, we mainly present the latest advancements in the design, synthesis, and catalytic properties of MOFs and COFs with inherent enzyme-mimicking activity in the past five years (Scheme 1). The general enhancement approaches of inherent enzyme activities are discussed by the structural characterization of MOFs and COFs. Finally, the sensing applications of MOF and COF nanozymes in combination with other analytical detection techniques are summarized, by which the intrinsic connection between the catalytic activity of the nanozymes and their recognizable detection signals is analyzed. Furthermore, we discuss the challenges and opportunities in the field of MOF and COF nanozyme sensors and provide insights into future directions.

## 2. Catalytic Mechanisms of MOF and COF Nanozymes

**Scheme 2:**
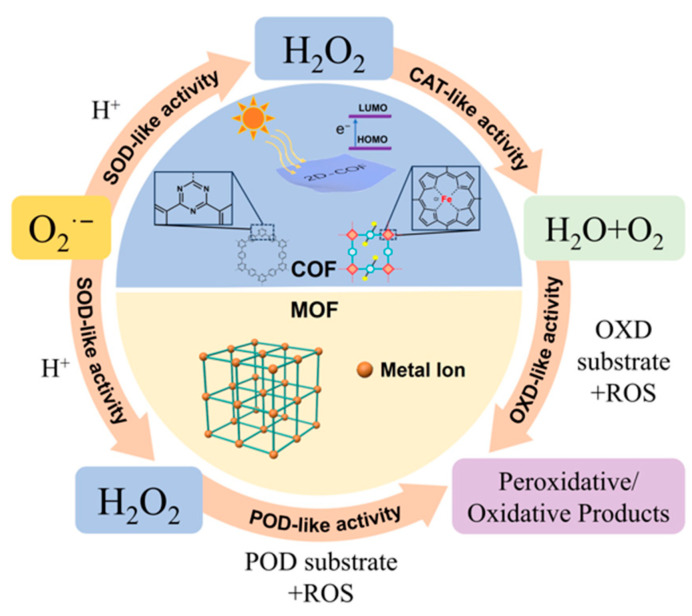
General catalytic mechanism of MOF and COF nanozymes.

The unique framework structure of MOFs and COFs gives them a large specific surface area and high porosity, which not only provides more adsorption catalytic sites, but also increases the diffusion rate of substrates and products. Scheme 2 discusses the sources of inherent enzyme activity of MOF and COF nanozymes and briefly analyzes their catalytic mechanisms.

Generally, MOFs are composed of inorganic metal centers and organic ligands connected to each other through self-assembly. Therefore, on the one hand, the enzyme-like catalytic activity of MOFs relies on the redox of metal sites. Multifarious MOF nanozymes based on Fe, Cu, Ce, Ni, etc., have been reported to possess enzyme-like activities [29,30,31,32]. Additional, various organic ligands and structures also provide MOF nanozymes with unique properties. Li’s group found that Cu/Zr bimetallic MOFs with 1H-pyrazole-4-carboxylic acid (H_2_Pyc) as a ligand contains trinuclear copper centers that mimic natural catecholoxidase active sites [33], which provides good specificity and high catalytic activity as a novel catechol oxidase nanozyme. Sha et al. grafted Cu^2+^ and a chiral His-coordinated copper onto Zr-based MOF-808 basic backbones to mirror the bimetal active site of natural catechol oxidase [34]. The synthesized MOF-L(D)-histidine-Cu was found to have even better catalytic selectivity over the chiral substrates than natural enzymes.

At present, the catalytic activity of COF nanozymes commonly originates from bionic sites, π-electron conjugation, or metal-containing monomers. The COF structure is rich in carbon and nitrogen, which form high-density bionic sites, and exhibits excellent enzyme-like activities. A research team reported that covalent triazine-based frameworks (CTFs) exhibit both peroxidase-like activity and oxidase-like activity, which can catalyze the oxidation of chromogenic substrates with or without H_2_O_2_ at very high efficiency levels [35]. The CTF contains a large number of C and N elements, which possess both peroxidase and oxidase activities. Its oxidase-like activity mostly stems from the generation of O_2_^−^, and peroxidase-like activity is derived from catalyzing the decomposition of H_2_O_2_ into ·OH. Some two-dimensional (2D) COFs have been shown to have high enzyme-mimicking activity due to a large specific surface area and periodic π-electron conjugation. Peng and colleagues proposed an ultrathin 2D COF called TTPA-COF with oxidase-mimicking activity that can be regulated by external light irradiation [36]. Because of the narrowband energy of TTPA-COF nanosheets, electrons are easily excited from HOMO to LUMO, and electron–hole pairs are generated. The photogenerated electrons are captured by O_2_ in the solution, resulting in an active substance, and the holes (h^+^) remain in the HOMO. Other COFs are constructed by combining metal-containing monomers with enzyme-like activity. Zhou et al. proposed a chiral COF nanozyme with Fe(III) meso-tetra (4-carboxyphenyl) porphine chloride (TCPP(Fe)) and L-histidine (L-his) [37]. The POD-like activity of the COF nanozyme was realized by Fe ions in TCPP(Fe), and the modification of L-his could further enhance the catalytic activity. The incorporation of L-his units also grants it selectivity for L-dopa in the oxidation of dopa enantiomers.

## 3. Nanozyme Types Based on MOFs and COFs

In recent years, people have been keen on the development and exploration of nanozymes and have developed a variety of oxidases (OXDs), peroxidases (PODs), superoxide dismutases (SODs), catalases (CATs), and hydrolases (HYDs) with different enzyme catalytic properties [8,38]. The unique framework structures of MOFs and COFs give them a large specific surface area and high porosity, which not only provides more adsorption catalytic sites but also increases the diffusion rate of substrates and products. Table 1 and Table 2 show some typical MOF and COF nanozymes with various types of enzyme activities, including their types and compositions, respectively. We will then discuss these nanozymes according to the differences in enzyme-like catalytic mechanisms.

### 3.1. Oxidases

Nanozymes based on MOFs and COFs can oxidize the substrate (electron donor) into the corresponding oxidation product in the presence of O_2_ (electron acceptor) and generate H_2_O or H_2_O_2_ [Equations (1)–(3)] [39]. It has been reported that the vast majority of MOFs exhibit excellent oxidase catalytic activity due to their large number of catalytic sites and metal centers (such as Cu, Fe, Zr, and Ce) [40,41,42,66]. Among these reported studies, Cu-based MOFs have attracted the most attention, followed by Fe-based and Ce-based MOFs. In the work of Li and Brondani, Cu-based MOFs were used to establish colorimetric and electrochemical detection methods of catechol, respectively [43,67]. In a recent study, a selective Cu-based MOF oxidase-like nanozymes for tryptophan and histidine processing was constructed based on the redox of copper species. The pocket formed by a tryptophan (Trp)/His and copper site structure can serve as a precise capture and specific catalytic center for oxidation, thereby achieving the selective detection of ascorbic acid from complex sweat [44]. Shen et al. prepared Cu-BTC MOFs at low temperature using 1,3,5-benzene tricarboxylic acid and Cu^2+^ ions. The Michaelis constant (K_m_) and maximum velocity (V_max_) for OPD were 0.0567 mmol/L, and 1.53 × 10^−8^ M/s. respectively, indicating that the prepared MOF nanozyme has strong affinity with o-phenylenediamine (OPD). Corma et al. found that after coating UiO-66(Ce) on the surface of defective CeO_2–x_ nanoclusters with terephthalic acid, terephthalic acid can promote charge transfer between metal nodes and realized that Ce^4+^/Ce^3+^ is converted into each other, thereby improving the enzyme catalytic activity [46].

AH + O_2_ → A + H_2_O (1)

AH + O_2_ + H_2_O → A + H_2_O_2_ (2)

(3) $$\mathrm{AH}+O_{2}\;\to \;A+O_{2}^{\cdot -}$$

COF-based oxidase-like nanozymes usually have low catalytic activity due to the lack of metal sites that provide redox capabilities. However, its interior is filled with a large number of π-conjugated frameworks, which is conducive to absorbing light, so using light as an external stimulus can significantly enhance the activity of COF nanozymes [62]. Li et al. combined the electron-rich functional unit Py and the electron-deficient functional unit TT to form a topologically ordered donor–acceptor Py-TT COF. This donor–acceptor COF has a narrow band gap and can significantly improve the absorption of visible light, promote the separation and transportation of photogenerated electron-hole pairs, generate a large amount of reactive oxygen species under oxygen and light, and then exhibit excellent photocatalytic oxidase activity [63]. Similarly, Lin et al. used the light-driven properties to enhance the oxidase activity of COFs. The photogenerated electrons in tetrakis(4-aminophenyl)ethane(ETTA)-4,4′-(thiazolo[5,4-d]thiazole-2,5-diyl)dibenzaldehyde (Tz) COFs can reduce O_2_ to $O_{2}^{\cdot -}$ and then oxidize 3,3′,5,5′-tetramethylbenzidine (TMB), OPD, and 2,2′-azino-bis (3-ethylbenzothiazoline-6-sulfonic acid (ABTS) to change color. In incorporating S^2−^ to inhibit photocatalytic oxidase activity, a light-triggered colorimetric sensing method was developed to detect S^2−^ [68].

### 3.2. Peroxidases

**Figure 1:**
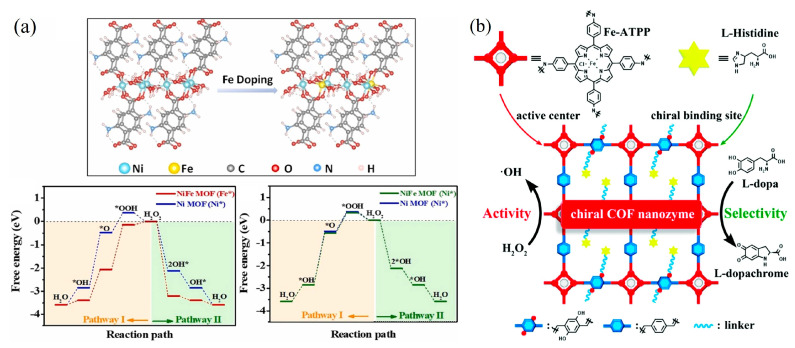
(**a**) Schematic of peroxidase catalysis based on Ni/Fe-MOFs, the catalytic sites (Fe, Ni) and intermediates were labeled with *. [32]. Copyright 2022, Elsevier. (**b**) Preparation and catalytic mechanism of chiral COF peroxidase [37]. Copyright 2020, Royal Society of Chemistry.

Peroxidase is an enzyme that uses H_2_O_2_ as an electron acceptor to catalyze the oxidation of substrates [Equations (4) and (5)]. In recent years, many MOF/COF-based nanozymes with peroxidase activity have been discovered. Fe-MOF is the most studied MOF nanozyme with peroxidase activity, such as MIL-88(Fe) [47], MIL-53(Fe) [48], etc., which exhibits excellent peroxidase properties through the redox of metal sites. Some other Cu-based MOFs [31], Ce-based MOFs [49], and Ni-based MOFs [50] are also used as simulated peroxidase materials to catalyze substrate oxidation. There is no doubt that, compared with common metal oxides and noble metal-based and C-based nanozymes, the large specific surface area and high porosity of MOFs can expose more nanozyme catalytic sites. In order to further improve the performance of MOF nanozymes, researchers introduced another metal into monometallic MOFs and successfully prepared a bimetallic MOF. Compared with monometallic MOFs, these bimetallic MOFs often exhibit higher synergistic effects in enhancing the catalytic performance of nanozymes. Huang et al. introduced Fe active sites into Ni-MOF through microwave-assisted methods. The enzyme activity of the synthesized Ni/Fe-MOF was 2.8 times higher than that of pure Ni-MOF (Figure 1a), and the affinity for H_2_O_2_ was increased to 42.5 times. DFT calculations show that the dissociation energy of H_2_O_2_ and the adsorption energy of reactive oxygen intermediates on the Fe site are more negative than those on the Ni site, thereby producing higher peroxidase activity [32]. Wang et al. synthesized Mn/Fe-MIL(53) by introducing Mn ions into Fe-MOF via a one-pot hydrothermal method, where Mn ions stimulated the oxidation cycle of Fe^2+^/Fe^3+^ and Mn^2+^/Mn^3+^ electron pairs. The synergistic enhancement of electron pairs further increased the electron transfer rate to generate more $O_{2}^{\cdot -}$, and a simple and sensitive strategy for the colorimetric detection of organophosphorus pesticides was established [48]. To improve the peroxidase activity of MOF nanozymes, it can also be achieved by changing its three-dimensional (3D) structure into a 2D structure [51,52]. Compared with 3D-MOFss, 2D-MOFs can quickly reduce mass transfer resistance during the catalytic reaction, effectively shortening the molecular diffusion distance of free radicals, and facilitate the substrate molecules to approach the active sites on the surface with a smaller diffusion barrier, thereby achieving enhanced catalytic performance [22]. The stripping of a 3D Cu-CAT to a 2D MOF through dextran assistance increases the specific surface area and exposes more catalytic sites, which greatly improves its peroxidase activity. Based on the fact that the functional group (-SH) in Acetylcholinesterase (AChE) can bind to the catalytic active sites of Cu-CAT nanosheets and reduce its peroxidase-like activity, a simple and highly sensitive colorimetric strategy was developed to monitor AChE levels [53].

AH + H_2_O_2_ → A + H_2_O (4)

AH + O_2_ + H_2_O → A + H_2_O_2_ (5)

Compared to MOF peroxidase, relatively little has been reported on COF peroxidase up to now due to the lack of an active catalytic center. He et al. first discovered the triazine-based COF nanozyme CTF, which has peroxidase and oxidase activities. In the presence of hydrogen peroxide or dissolved oxygen, the CTF can not only decompose H_2_O_2_ into ·OH but also catalyze dissolved oxygen to generate ·OH, causing substrates TMB and OPD to oxidize and change color [35]. Qu et al. reported a COF nanozyme that can be used for chiral recognition, and the selective recognition of COF was controlled by changing the doped amino acids, where its enzyme activity was mainly dependent on the synergistic interaction between the doped amino acids and the metal ions in the iron porphyrin. The incorporation of L-his makes the COF nanozyme selective for the L-dopa oxidation of dopa enantiomers with a selectivity factor of 1.86 (Figure 1b), while the use of d-His enables the COF nanozyme to reverse the oxidation of L-dopa to D-dopa [37]. This study opened up a new way for the selective oxidation of nanozymes.

### 3.3. Superoxide Dismutase

Superoxide oxidase is mainly used to remove excess $O_{2}^{\cdot -}$ in the body and can catalyze $O_{2}^{\cdot -}$ to generate O_2_ and H_2_O_2_ to maintain the redox balance in the organism [Equation (6)]. Recently, Li et al. found that Sn-based metalloporphyrin can effectively catalyze the disproportionation of superoxide radical anions into hydrogen peroxide and oxygen. The Sn^4+^ in the porphyrin center first combines with $O_{2}^{\cdot -}$ to undergo electron transfer, and Sn^4+^ is reduced to generate O_2_, and then Sn^2+^ is oxidized by another $O_{2}^{\cdot -}$ with the production of H_2_O_2_, completing the Sn^4+^/Sn^2+^ redox [54].

(6) $$2H^{+}+2O_{2}^{\cdot -}\;\to \;H_{2}O_{2}+O_{2}$$

### 3.4. Catalase

Catalase, which scavenges H_2_O_2_ from organisms and produces O_2_ and H_2_O_2_, has been extensively studied in the field of photodynamic therapy [Equation (7)]. In general, MOFs and COFs with hydrolase activity also possess other enzyme-like activities, such as bimetallic porphyrin-based COFs constructed through a Schiff base reaction, for which its unique topology and bimetallic active sites synergistically enhance OXD, POD, and CAT activities [64]. Zhang et al. prepared an Fe-MOF single-atom nanozyme (SAzyme) based on a mechanically assisted in situ MOF encapsulation strategy, and the catalase and POD activities of the Fe-MOF were significantly enhanced by adjusting the distances between neighboring atoms in the single iron sites [55]. In addition, the cascade action of SOD enzymes and CAT enzymes is a common antioxidant pathway. Zhang et al. developed a bimetallic nanozyme Cu-TCPP(Mn) by embedding manganese and copper into porphyrin, which has dual enzyme activities of SOD and CAT. Cu-TCPP(Mn) first converts oxygen free radicals into H_2_O_2_, and then H_2_O_2_ is decomposed into harmless O_2_ and H_2_O, which can protect heart tissue from damage caused by oxidative stress and inflammation [69].

2H_2_O_2_ → O_2_ + 2H_2_O (7)

### 3.5. Hydrolase

**Figure 2:**
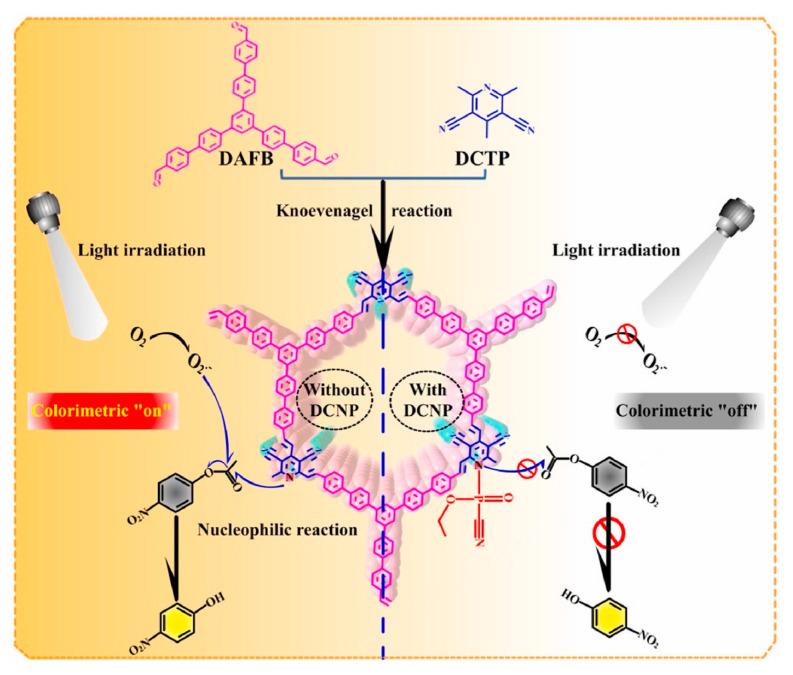
The preparation of COF hydrolase and the source of light-enhanced COF hydrolase activity [65]. Copyright 2023, Elsevier.

The hydrolysis enzyme is a general class of enzymes that catalyze hydrolysis reactions, mainly using water as a receptor for the transfer group to catalyze the hydrolysis reaction. Hu et al. prepared a green amino acid Zr-MOF using L-aspartic acid and water as ligands, and the catalytic activity of phosphatase hydrolysis was higher than that of ZrCl4 and Zr(OH)_4_, with a large number of Zr-OH groups in Zr-MOF playing a key role in substrate binding and hydrolysis [56]. Based on the hydrolase action mechanism of MOFs, the preferred active site is a high-valent Lewis acid metal ion, which can more easily activate the carbonyl group or phosphorus group in the substrate, thereby increasing the electrophilicity and reactivity of the central carbon or phosphorus. Through data screening, Wei’s team fine-tuned the Lewis acidity of metal clusters and shortened the ligand length to increase catalytic active sites [57]. Finally, the Ce-FMA-MOF hydrolase prepared with high-priced Ce^4+^ and fumaric acid was able to cleave phosphate bonds, amide bonds, and glycosidic bonds. On the other hand, the lack of enzyme catalytic sites in COFs makes it crucial to introduce catalytic sites into COFs to make them enzyme-catalytic. The catalytic activity of the pyridine group can be integrated into the donor–acceptor (D-A) conjugated skeleton through the Knoevenagel condensation reaction, yielding the DAFB-DCTP COF hydrolysis and photosensitivity properties (Figure 2). A colorimetric detection strategy for organophosphorus nerve agent simulants was constructed by light-enhanced COF hydrolysis activity on p-nitrophenyl acetate [65].

### 3.6. Multifunctional Enzymes

**Figure 3:**
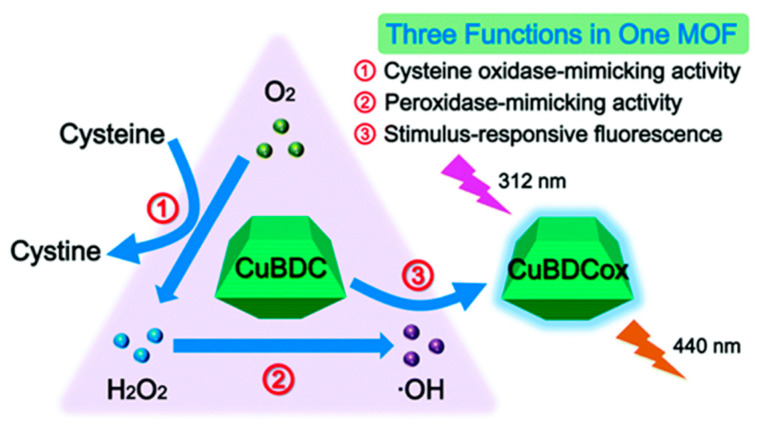
Schematic of the cascade reaction based on the Cu-BDC multifunctional enzyme [59]. Copyright 2018, Royal Society of Chemistry.

At present, it has been found that some MOFs and COFs have multiple enzyme activities, which can give them higher catalytic efficiency and multifunctional analytical sensing. For instance, Li et al. constructed Cu-BDC with cysteine (Cys) oxidase and peroxidase activities using terephthalic acid as a ligand. Under light stimulation (Figure 3), Cu-BDC first oxidizes cysteine to generate H_2_O_2_ in the presence of dissolved oxygen and then catalyzes the generation of ·OH under its own peroxidase activity. ·OH can oxidize the Cu-BDC ligand to produce a fluorescent signal. The trinity Cu-BDC creates a convenient and fast sensing platform for the determination of Cys [59]. Bai et al. constructed porphyrin-based COF nanozymes through Schiff base reaction. The unique topological structure and bimetallic active sites give it the three enzyme activities of OXD, POD, and CAT [64]. However, obtaining MOFs and COFs with multiple enzyme activities often requires metals [58,70] or natural enzymes [29,71] to be encapsulated between organic frameworks, which puts more stringent requirements on the development of multi-active nanozymes. In order to solve this problem, Zhao et al. used a high-temperature gas migration strategy by placing ZIF-8 and iron powder in a ceramic boat under nitrogen flow, respectively, and Fe atoms were combined with pyrolytically volatilized Zn sites to obtain Fe-MOF SAzymes enriched with a large number of defects. Systematic studies of these SAzymes revealed that the large number of Fe-N_4_-enriched sites confer the Fe-MOF SAzymes with POD-, OXD-, CAT-, and GPx-catalytic activities [72]. Liang et al. prepared Ce-BPyDC MOFs using a simple hydrothermal method that retains the coexistence of Ce^3+^ and Ce^4+^ in the MOF structure, establishes a Ce^3+^/Ce^4+^ redox cycle inside, and gives the Ce-MOF the activities of oxidase and peroxidase [60].

## 4. Sensing Analysis Based on MOF and COF Nanozymes

Nanozymes have many remarkable properties that provide great possibilities for the design of sensor analysis. In general, MOF and COF nanozymes can realize the trace analysis of target substances mainly in the following two ways: First, enzyme substrates (H_2_O_2_, etc.) and substrate-related molecules (such as glucose) that can be directly detected have significant signal changes before and after nanozyme catalysis; second, the detection of such substances is achieved by regulating the activity of nanozymes through external molecules to cause signal increase or decrease. According to previous reports, the sensing applications of MOF and COF nanozymes mainly include colorimetric, fluorescence, chemiluminescence, electrochemical, and SERS sensors. Next, we will analyze these sensing principles in detail. Table 3 summarizes the typical sensors based on MOF and COF nanozymes in the past five years.

### 4.1. Colorimetric Sensing

**Figure 4:**
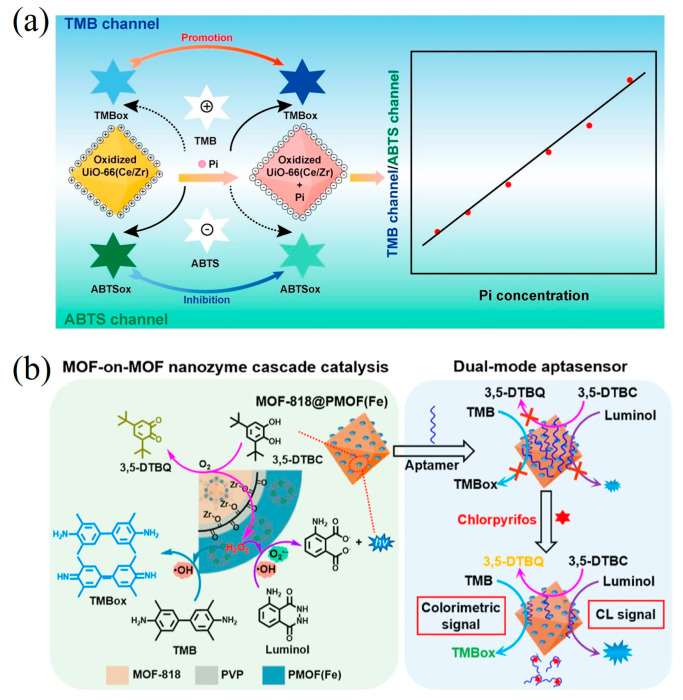
(**a**) Two-scale colorimetric sensing of phosphate ions induces changes in UiO-66 (Ce/Zr) differential oxidase activity [39]. Copyright 2020, Elsevier. (**b**) MOF-on-MOF nanozyme cascade catalytic mechanism and aptamer-based dual detection construction [75]. Copyright 2023, American Chemical Society.

Colorimetric analysis, as one of the most typical detection methods, is an efficient and rapid analysis with target detection using the naked eye or portable equipment to identify the color change in the solution, which has been widely used in the fields of environmental pollution monitoring, food safety monitoring, and biochemical analysis [96,97]. The principle of colorimetric sensing is to identify the target analyte, perform quantitative analysis based on the target analyte content and the color change in the solution, and transfer electrons between substrates to catalyze specific chemical reactions and produce corresponding signal changes. Based on this characteristic, nanozymes can be used as signal markers to detect target analytes, converting the amount of target analytes into signals that can be quantitatively analyzed for measurement, that is, oxidizing colorless substrates to colored substrates, thereby achieving the purpose of visual detection. Compared with colorimetric sensors without nanozymes, the combination of nanozymes and colorimetry plays a role in signal amplification to a certain extent, can produce obvious color and signal changes, significantly reduces the detection time, and helps improve the detection performance of colorimetric sensors and achieve rapid detection. In recent years, nanozymes based on MOFs and COFs have achieved indirect colorimetric detection of various target substances such as glucose, ascorbic acid, glutathione, organophosphorus pesticides, etc., by catalyzing the color change in substrates (TMB, OPD, ABTS) [20,98,99]. Li et al. prepared Mo/Cu-2-MIN nanozyme by doping Mo in Cu-2-MIN MOF and used TMB as a colorimetric substrate. They found that Mo/Cu-2-MIN exhibited higher peroxidase-like activity than pure Cu-2-MIN and attributed this improvement to the fact that Mo as a co-catalyst accelerated electron transfer in the system, which helped generate reactive oxygen species (ROS) and thus improved the catalytic activity [100]. Cai et al. demonstrated that the defects also accelerate the electron transfer in the nanozyme catalytic process. Defect-rich MIL-101(Fe)-OH-D was obtained by etching MIL-101(Fe), which showed a colorimetric detection range of 5–300 ng/mL for dichlorvos [78]. Conversely, by inhibiting the catalytic activity of MOF nanozymes has also been reported. Typically, aptamers adsorbed on the surface of MOFs with a high specific surface area inhibit the enzyme-like activity of MOFs, and specific binding between the aptamers on the surface, and subsequently added targets trigger the release of rigid complexes on the surface of MOFs to reactivate the enzyme-like activity. Through this aptamer-controlled inhibition–activation mechanism of nanozyme activity, Liu et al. proposed a colorimetric assay for marine biotoxins based on Zr and Fe bimetallic MOFs [61]. The coordinated action of the bimetals endowed the Zr/Fe-MOF with high peroxidase-like activity, and the enzyme-like activity was controlled by the sequential incorporation of tetrodotoxin aptamer and tetrodotoxin, with a LOD of tetrodotoxin as low as 0.07 ng/mL and an assay range of 0.1–200 ng/mL. MIL-88A(Fe) prepared by Wang et al. possessed peroxidase-like activity, which could significantly catalyze the oxidative bluing of TMB, and the catalytic activity was inhibited under the action of thrombin aptamer, and the detection limit of thrombin based on the obvious colorimetric change was as low as 0.8 nmol/L [73]. The advantage of this method is that it is highly specific and effective only for the target, which is suitable for the qualitative and low concentration detection of specific targets. Luo et al. prepared Mn/Fe-MIL(53) by introducing Mn ions into Fe-MIL(53) via a hydrothermal method, which not only enhanced the oxidase activity of MOFs but also endowed the material with the property of being specifically destroyed by choline [48]. Accordingly, a selective strategy for the detection of organophosphorus pesticides was established, with the detection ranges of 10–120 nmol/L, and 5–50 nmol/L, for methyl parathion and chlorpyrifos, respectively. The peroxidase activity of NH_2_-Cu-BDC is also inhibited by choline, and the detection range of chlorpyrifos was 1.8–180 ng/mL with a low detection limit of 1.57 ng/mL [74]. Interestingly, Li et al. proposed a dual-channel proportional colorimetric strategy based on analyte-triggered oxidized UiO-66(Ce/Zr) in the detection of phosphate ions [39]. Phosphate ions present in solution adsorbed on the surface of oxidized UiO-66(Ce/Zr) through Zr-O-P bonds change the surface charge of oxidized UiO-66(Ce/Zr), which significantly promotes the oxidation of TMB and inhibits the oxidation of ABTS (Figure 4a). The highly sensitive detection of phosphate ions with a detection limit as low as 1.1 μmol/L was achieved by integrating the two chromogenic reactions. In a recent study, Zhang et al. integrated MOF-818 with catechol-like oxidase activity and iron porphyrin MOF with peroxidase-like activity to prepare MOF-818@PMOF(Fe) [75]. MOF-818 catalyzed the in situ production of H_2_O_2_ from catechol substrates, and PMOF(Fe) catalyzed the production of reactive oxygen species from H_2_O_2_, which resulted in the chromogenic luminescence of TMB and luminal (Figure 4b). In addition, a colorimetric/chemiluminescent bimodal aptamer for chlorpyrifos was successfully constructed using this cascade reaction, and the detection limit was as low as 0.26 ng/mL.

For COF nanozymes, due to the lack of variable-valence metals as catalytic sites, their development in various fields lags behind that of MOF nanozymes. Studies have shown that COFs have photoactive catalytic ability due to their π-conjugated structure and adjustable band gap, which can absorb light to achieve intramolecular electron transfer and free radical formation, and they exhibit light-regulated nanozyme activity. For example, Li et al. constructed an ETTA-Tz nanozyme with photoexcitation properties using electron-deficient Tz and electron-rich ETTA as structural units [68]. The photogenerated electrons can reduce dissolved oxygen to superoxide radicals and then oxidize the substrate, and the catalytic activity of the ETTA-Tz nanozyme can be directly controlled by turning on/off the light to turn on the catalytic activity of ETTA-Tz nano-enzymes, which has successfully achieved a highly sensitive detection of S^2−^, with a detection range of 1–50 μmol/L and detection limit of 0.27 μmol/L. COF-366/VO prepared based on vanadium docked porphyrin, the inherent π-conjugated structure, is excited to generate photogenerated charge carriers under illumination, and the photogenerated electrons of COF-366 can be transferred to VO, further improving the photogenerated electricity [79]. The separation of charge carriers produces a large number of free radicals that oxidize OPD into yellow oxidized OPD (OPDox). A colorimetric sensing strategy of L-arginine (L-arg) was established based on its inhibitory effect on COF-366/VO enzyme activity. Reports on photoactive MOF nanozymes are relatively rare, and only three forms of photoactive MOF nanozymes (2D, 3D, and adenine-TCPP) prepared by Liu et al. using the photosensitizer TCPP as a ligand have been found to exhibit excellent photoactive oxidase activity under the light condition, which can be used for the colorimetric sensing of various antioxidants [76].

### 4.2. Fluorescent Sensing

**Figure 5:**
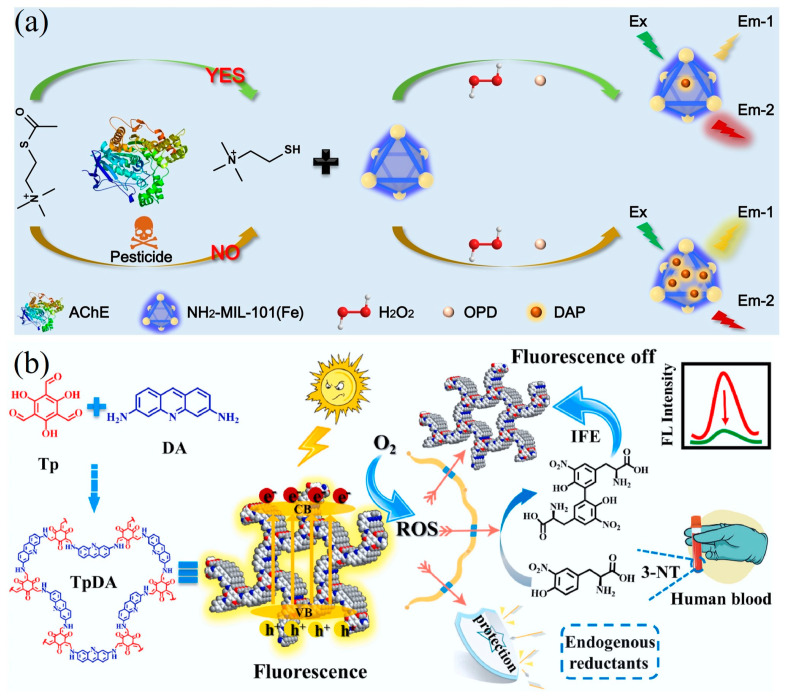
(**a**) Schematic representation of ratiometric fluorescent nanosensor based on bifunctional NH_2_-MIL-101(Fe) for pesticide detection [83]. Copyright 2021, Elsevier. (**b**) Construction of multifunctional TpDA COF nanozyme for label-free 3-NT detection [84]. Copyright 2023, Elsevier.

Fluorescence sensing based on optical sensors achieves highly sensitive sensing detection through fluorescence quenching or fluorescence enhancement mediated by the target analyte [101]. Free radicals generated during the catalytic process of nanozyme fluorescence sensing platforms usually cause photoinduced electron transfer (PET), resulting in the fluorescence enhancement or fluorescence quenching of the target analyte [102,103]. Otherwise, fluorescence resonance energy transfer (FRET) between the nanozymes and the substrate can also lead to the blue shifting or red shifting of the fluorescence signal in the detection system [104,105]. In addition, the ligands, metal centers, or structural units inside the MOF and COF nanozymes can also be used as fluorescent probes to interact with the target analyte to produce obvious fluorescence signal changes and can effectively simplify experimental operations and improve detection sensitivity. In recent years, this self-induced MOF- and COF-based nanozyme fluorescence sensor has received widespread attention [106,107]. For example, Li et al. used Cu-BTC to catalyze the oxidation and polymerization of OPD, and the resulting polymer nanoparticles have a high photothermal effect [80]. AChE inhibits the catalytic activity of Cu-BTC and changes the fluorescence and photothermal dual signals of the OPD cascade polymer to achieve the dual-mode indirect detection of AChE activity. The detection ranges in the two modes are 0.2–40 U/L and 0.5–50 U/L, respectively. Liu et al. developed NH_2_-MIL-101(Fe) for pesticide detection based on a bifunctional iron-based MOF (Figure 5a), where the Fe site inhibits the fluorescence intensity of the MOF at 428 nm by catalyzing OPD oxidation, and the presence of pesticide residues can further increase the generation of OPDox and further reduce the fluorescence signal, which can sensitively detect carbaryl in the range of 2–100 ng/mL with a detection limit of 1.45 ng/mL [83]. In a recent study, Liang et al. constructed a multifunctional COF nanozyme (TpDA) with light-responsive and fluorescent properties using 1,3,5-triformylphloroglucinol (Tp) and 3,6-diaminoacridine (DA) as structural units for the sensitive detection of 3-nitrotyrosine (3-NT) [84]. Under light irradiation, 3-NT was first oxidized by the free radicals generated during the catalysis of TpDA nanozyme. Then, due to the internal filter effect mechanism, the fluorescence signal of TpDA is quenched through the oxidation product of 3-NT, and the fluorescence intensity decreases with the concentration of 3-NT (Figure 5b). This shows a downward trend and has a good linear relationship with 3-NT within a certain concentration range (0.05~80 μmol/L), with a detection limit as low as 0.011 μmol/L.

### 4.3. Chemiluminescent Sensing

Chemiluminescence sensing has high sensitivity and is one of the important means of trace analysis. It has been widely used in environmental monitoring, clinical analysis, biochemistry and other fields, such as pollutant determination, immunoassay, and trace metal analysis [108,109]. Chemiluminescent sensing mainly relies on the redox reaction of the H_2_O_2_–luminol system to generate chemiluminescent signals, and then the luminescence intensity is converted into a digital signal through a converter. Similar to colorimetric sensing, MOF- and COF-based nanozymes with peroxidase catalytic activity can significantly increase the chemiluminescent signal intensity and detection sensitivity by catalyzing the H_2_O_2_–luminol system and have been widely used to construct nanozyme chemiluminescent sensors. Based on the above chemiluminescence system, Wang et al. used iron porphyrin to prepare a single-atom MOF-FeP nanozyme, for which its peroxidase-like activity enables it to catalyze the chemiluminescence of luminol substrates [85]. By integrating MOF-FeP into conventional strips, a rapid and highly sensitive Epstein–Barr virus (EBV)-IgAs detection tool was created. The MOF-FeP test strip can detect three EBV-IgAs simultaneously in only 16 min, greatly improving the accuracy and detection time of EBV-related nasopharyngeal cancer screening. Similarly, Li et al. constructed, for the first time, a flow injection chemiluminescence immunoassay (FI-CLIA) based on a Fenton-like activity single-atom cobalt nanozyme (Co SAzyme) for the rapid and sensitive detection of serum 5-fluorouracil (5-Fu) [86]. Co SAzyme uses ZIF-8 MOFs as the core, which can catalyze the decomposition of H_2_O_2_ to generate a large amount of superoxide radical anions, thereby effectively amplifying the chemiluminescent luminol–H_2_O_2_ system. Under optimal conditions, the detection range of 5-Fu is 0.001–1000 ng/mL, and the detection limit is 0.29 pg/mL (S/N = 3). Recently, Zeng et al. constructed a flow injection chemiluminescent immunoassay for FF (florfenicol) in food residues based on Ni/Co-MOF peroxidase. Ni/Co-MOF first formed a stable immune probe with the florfenicol antibody. According to the principle of competitive immunity, FF competed with the coated antigen on the carboxyl resin beads for the limited binding sites on the FF antibody, changing the peroxidase activity and quantitatively analyzing FF based on the signal change. Under the optimal experimental conditions, the detection range of FF was 0.0001–1000 ng/mL, and the detection limit (LOD) was 0.033 pg/mL [87].

### 4.4. Electrochemical Sensing

In recent years, electrochemical detection has developed rapidly in environmental monitoring, chemical identification, and food safety testing due to its advantages of low cost, speed, high sensitivity, and the ability to achieve online monitoring [32,110]. Electrochemical sensing is realized by modifying the recognition unit corresponding to the target analyte on the surface of the sensing electrode, generating an oxidation or reduction reaction and then outputting an electrical signal [111,112,113]. The intensity of the electrical signal is closely related to the intensity of oxidation or reduction of the target analyte on the electrode surface. By modifying the nanozyme on the electrode surface, it can be used as a generator of electrically active probe or signal amplification tag to obtain highly sensitive electrochemical signals through the oxidation or reduction of the substrate in the catalytic reaction system, which can be used to realize the construction of nano-enzymatic electrochemical sensors. For example, Gao et al. modified Ni-HHTP with peroxidase activity on a screen-printed electrode and non-covalently adsorbed the tetracycline aptamer (TC-apt), which unexpectedly improved the catalytic performance of the nanozyme. After the interaction between TC and the TC-apt, the TC-apt disengages and reduces the electrochemical signal. This principle is used to complete the quantitative analysis of tetracycline from 10 pmol/L to 1.0 μmol/L, with a detection limit of 1.9 pmol/L [88]. Ma and colleagues assembled tyrosinase (Tyr) layer by layer in ultrathin copper porphyrin MOF nanofilms (Tyr@Cu-TCPP) to construct an electrochemical biosensor for bisphenol A detection. Tyr@Cu-TCPP nanozyme not only has long-term storage stability but also maintains good enzyme activity in strong acid–base environments and high temperatures [89]. It is worth noting that Hu et al. achieved the highly sensitive detection of pathogenic Staphylococcus aureus using a Cu-TCPP(Fe) nanozyme [90]. Through anchoring Staphylococcus aureus on the GCE electrode surface modified with vancomycin and anti-Staphylococcus aureus antibodies through a dual recognition strategy, 2D Cu-TCPP(Fe) can effectively catalyze the oxidation of OPD to generate OPDox; OPDox is electrochemically reduced to obtain the reduction current, and the Staphylococcus aureus concentration corresponds to the peak current. Li et al. prepared a new three-layer iron-based MOF (MIL-88@Pt@MIL-88) with intrinsic peroxidase properties by regulating the structure, and performed the ultra-sensitive detection of exosomal microRNA through a primer exchange reaction. Due to the inherent enzyme-like activity of MIL-88@Pt@MIL-88, it can effectively catalyze the decomposition of H_2_O_2_ to generate electrochemical signals for exosomal microRNA-21 sensing. Signal amplification is achieved with the help of a primer exchange reaction. MicroRNA-21 has a wide detection range from 1 f mol/L to 1 nmol/L, with a detection limit as low as 0.29 fmol/L [91].

### 4.5. Surface-Enhanced Raman Scattering Sensing

Surface-enhanced Raman scattering has the advantages of rich molecular information, high sensitivity, and a fast response. It can achieve the ultra-sensitive detection of trace molecules and has been successfully used in biological, food, environmental, and clinical sensing [114,115,116]. The inherent advantages of SERS such as fingerprinting, rapid detection, and portability can be extremely convenient for critical technologies. However, the small Raman cross-sectional area of many target molecules (e.g., glucose, lactate, and proteins) or a lack of Raman activity severely limits the development of SERS for analytical sensing. Recent studies have shown that the combination of nanozymes and SERS technology, using target molecules to stimulate the nanozyme substrate to produce significant changes in SERS signals, which can be used to realize the indirect detection of the target molecules, can help to establish and expand the integrated nanozymes and SERS platform for the sensing analysis.

**Figure 6:**
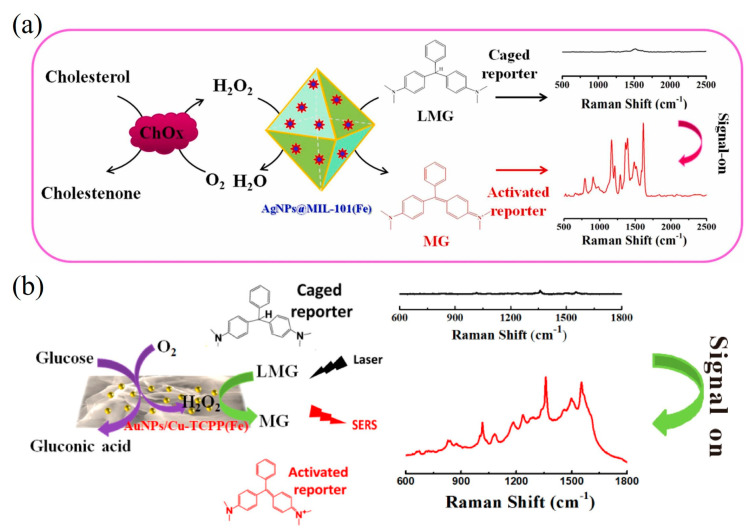
(**a**) Illustration of AgNPs@MIL-101(Fe)-based SERS biosensor used to detect cholesterol [93]. Copyright 2022, Elsevier. (**b**) Schematic of the enzyme-free tandem reaction strategy for SERS detection of glucose [95]. Copyright 2020, American Chemical Society.

Loading metal nanoparticles on the MOF surface can significantly enhance the SERS signal of the analyte molecules through the electromagnetic field enhancement mechanism and results in extremely high detection sensitivity. Wei et al. synthesized highly porous a AuNPs@MIL-101 nanozyme through in situ growth. Its excellent enzyme activity can oxidize the Raman inactive reporter leuco malachite green into active malachite green to generate SERS signals. Combined with native enzymes, the linear range of glucose and lactate in vitro is 10 to 200 μmol/L, with detection limits of 4.2 and 5.0 μmol/L, respectively [92]. He et al. grew Ag nanoparticles on the surface of MIL-101(Fe) and proposed a highly sensitive and specific sensing platform to monitor changes in cholesterol content based on its excellent peroxidase-mimicking activity and high SERS enhancement (Figure 6a) [93]. In a recent study, Ma et al. developed a nanozyme SERS sensor based on aptamer recognition for histamine detection. Histamine (HA) apts were modified on MIL-100(Fe)@AuNPs, cDNA was modified on the surface of AgNPs, and self-assembly was completed under the action of base complementary pairing, so that the SERS signal of oxidized TMB (TMBox) in the catalytic system was significantly amplified [94]. When HA is present, the strong affinity of HA for HA apt destroys the SERS amplification system, leading to a reduction in SERS intensity, and thus realizing the ultrasensitive detection of trace amounts of HA. In order to further improve the catalytic performance of MOF nanozymes, an effective strategy is to design the three-dimensional MOF structure into two-dimensional MOF nanosheets. Hu et al. grew highly stable AuNPs on Cu-TCPP(Fe) by in situ reduction and successfully synthesized the AuNPs/Cu-TCPP(Fe) nanozyme with cascade catalysis [95]. In the cascade-induced catalytic reaction, glucose can be catalytically oxidized by AuNPs to generate H_2_O_2_ (Figure 6b). The in situ generated H_2_O_2_ oxidized leuco-malachite green (LMG) to malachite green (MG) under the action of Cu-TCPP(Fe) and produced SERS signals, thus establishing a rapid detection method for glucose.

## 5. Challenges and Perspectives

In this review, we highlighted recent advances in MOF and COF nanozymes and presented their applications in analysis and detection. MOF and COF nanozyme sensors have been used as substitutes for natural enzymes in environment, food, medicine, and other fields. While great efforts have been put into MOF and COF nanozymes, they still face certain challenges:

Compared to natural enzymes, nanozymes may lack the precis selectivity for certain substrates. While they can catalyze a wide range of reactions, their selectivity might not match that of biological catalysts. To overcome this problem, nanozymes with favorable selectivity such as molecularly imprinted nanozymes or structural biomimetic nanozymes have been proposed. At present, the enzyme-like activities of nanozymes are mainly simulated redox enzymes and hydrolases. Exploring new catalytic activities is a necessary way to broaden the types and application scenarios of MOF and COF nanozymes. Developing more types of nanozymes and studying their catalytic mechanisms will help us to establish more diversified analytical methods. While large quantities of MOF and COF nanozymes have been proposed, studies on the catalytic mechanism of nanozymes are still insufficient, especially for COF-based nanozymes. The in-depth analysis of the catalytic mechanism of nanozymes, discovering the whole process of the enzyme-like reaction catalyzed by nanozymes from the molecular level, is crucial for the rational design and synthesis of high-performance nanozymes in the future. At present, most of the nanozyme sensors based on MOFs and COFs need to realize the indirect sensing of targets with signal substrates, which is not conducive to their convenience and accuracy. There is a need to develop more nanozyme sensors that can directly detect the signal of the target.

The applications on metal–organic framework and covalent–organic framework nanozymes open exciting avenues across biocatalysis, biosensing, and environmental remediation. Nanozymes with stronger selectivity, higher catalytic activity, and a greater variety of enzyme-like activity types are being rapidly proposed. Despite the challenges, research on nanozymes continuously offers boundless possibilities in solving catalysis problems.

## Figures and Tables

**Table 1:** Some typical MOF nanozymes and their enzyme-like activities.

Materials	Metal	Ligand	Enzyme-Like Activities	Ref.
UiO-66	Ce; Zr	terephthalic acid	OXD	[39]
NH_2_-Fe-BDC	Fe	2-aminoterephthalic acid 2-aminoterephthalic acid	OXD/POD	[40]
NH_2_-Cu-BDC	Cu	2-aminoterephthalic acid	POD	[41]
ZIF-67(Co)	Co	2-methylimidazole	OXD/POD	[42]
HKUST-1	Cu	benzene-1,3,5-tricarboxylic acid	OXD	[43]
STAM-17-OEt	Cu	5-ethoxyisophthalic acid	OXD	[44]
Cu-BTC	Cu	benzene-1,3,5-tricarboxylic acid	OXD	[45]
UiO-66	Ce	terephthalic acid	OXD	[46]
MIL-88(Fe)	Fe	terephthalic acid	POD	[47]
MIL-53(Mn/Fe)	Mn; Fe	terephthalic acid	OXD	[48]
Cu-TCPP(Fe)	Cu	Fe(III) meso-tetra (4-carboxyphenyl) porphine chloride	POD	[30]
NH_2_-Cu-BDC	Cu	2-aminoterephthalic acid	OXD	[31]
Ce-BTC	Ce	trimesic acid	POD	[49]
MIL-53(Ni)	Ni	p-benzenedicarboxylic acid	POD	[50]
NH_2_-Ni/Fe-BDC	Ni; Fe	2-aminoterephthalic acid	POD	[32]
Ni-TCPP(Fe)	Ni	Fe(III) meso-tetra (4-carboxyphenyl) porphine chloride	POD	[51]
Zn-TCPP(Fe)	Zn	Fe(III) meso-tetra (4-carboxyphenyl) porphine chloride	POD	[52]
Cu-CAT-HHTP	Cu	2,3,6,7,10,11-hexahydroxytriphenylene hydrate	POD	[53]
Sn-PCN222	Zr	Fe(III) meso-tetra (4-carboxyphenyl) porphine chloride	SOD	[54]
Fe-SAzymes	Fe	ZIF-8	POD/CAT	[55]
MIP-202(Zr)	Zr	l-aspartic acid	HYD	[56]
Ce-FMA	Ce	Fumaric acid	HYD	[57]
Fe/Zr-TPY	Fe; Zr	4′-(4-carboxyphenyl)[2,2′:6′,2″-terpyridine]-5,5″-dicarboxylic acid	POD	[58]
Cu-BDC	Cu	terephthalic acid	POD	[29]
Cu-BDC	Cu	p-benzenedicarboxylic acid	OXD/POD	[59]
Ce-BPyDC	Ce	2,2′-bipyridine-5,5′-dicarboxylicacid	POD	[60]
NH_2_-Fe/Zr-BDC	Fe; Zr	2-aminoterephthalic acid	POD	[61]

**Table 2:** Some typical COF nanozymes and their enzyme-like activities.

Materials	Building Unit 1	Building Unit 2	Enzyme-Like Activities	Ref.
FPY-COF	Linear dialdehydes	6-(4-formylphenyl) nicotinaldehyde	OXD	[62]
Py-TT COF	1,3,6,8-tetrakis(4-aminophenyl)pyre	thieno[3,2-b]thiophene-2,5-dicarbaldehyde	OXD	[63]
CTF	1,4-dicyanobenzene	1,4-dicyanobenzene	OXD/POD	[35]
Fe-COF	Fe(III) meso-tetra (4-carboxyphenyl) porphine chloride	Terephthaldehyde	POD	[37]
Fe/Co-COF	Fe/Co(III) meso-tetra (4-carboxyphenyl) porphine chloride	Terephthaldehyde	OXD/POD/CAT	[64]
DAFB-DCTP COF	4-[4-[3,5-bis[4-(4-formylphenyl)phenyl]phenyl]phenyl]benzoic acid	2,4,6-trimethylpyridine-3,5-dicarbonitrile	HYD	[65]

**Table 3:** Applications of MOF and COF nanozymes in analytical sensing.

Method	Materials	Analyte	Enzyme-Like Activities	Linear Range	LOD	Ref.
Colorimetry	Cu-BDC	Glucose	POD	10–500 μmol/L	4.1 μmol/L	[29]
	NH_2_-Fe/Zr-BDC	Tetrodotoxin	POD	0.1–200 ng/mL	0.07 ng/mL	[61]
	MIL-88A(Fe)	Thrombin	OXD	10–80 nmol/L	0.8 nmol/L	[73]
	Mn/Fe-MIL(53)	Methyl parathion Chlorpyrifos	OXD	10–120 nmol/L 5–50 nmol/L	2.8 nmol/L 0.95 nmol/L	[48]
	NH_2_-Cu-BDC	Chlorpyrifos	POD	1.8–180 ng/mL	1.57 ng/mL	[74]
	UiO-66(Ce/Zr)	Phosphate ions	OXD	3.3–666.7 μmol/L	1.1 μmol/L	[39]
	MOF-818@PMOF(Fe)	Chlorpyrifos	POD OXD	10–40 ng/mL	0.26 ng/mL	[75]
	Zn-TCPP	Multiple antioxidants	OXD	-	-	[76]
	Cu-TCPP(Fe)	Dichlorophen	POD	4–130 μmol/L	1.33 μmol/L	[77]
	MIL-101(Fe)-OH-D	Dichlorvos	POD	5–300 ng/mL	2.06 ng/mL	[78]
	ETTA-Tz COF	S^2−^	OXD	1–50 μmol/L	0.27 μmol/L	[68]
	COF-366/VO	L-Arginine	OXD	8.1–330 μmol/L	3.5 nmol/L	[79]
Fluorescent	Cu-BTC	AChE	OXD	0.2–40 U/L	0.03 U/L	[80]
	Tb-IDA-Fe_3_O_4_	Tyrosine	POD	0–10 μmol/L	0.1 μmol/L	[81]
	Tb-OBBA	17β-estradiol	POD	0–100 nmol/L	50 pmol/L	[82]
	NH_2_-MIL-101(Fe)	Carbaryl	POD	2–100 ng/mL	1.45 ng/mL	[83]
	TpDA COF	3-NT	OXD	0.05–80 μmol/L	0.011 μmol/L	[84]
Chemiluminescent	MOF-FeP	EBV-IgAs	POD	-	-	[85]
	ZIF-8	5-Fluorouracil	POD	0.001–1000 ng/mL	0.29 pg/mL	[86]
	Ni/Co-BTC	Florfenicol	POD	0.0001–1000 ng/mL	0.033 pg/mL	[87]
Electrochemical	Ni-HHTP	Tetracycline	POD	10 pmol/L–1.0 μmol/L	1.9 pmol/L	[88]
	Tyr@Cu-TCPP	Bisphenol A	CAT	3.5 nmol/L–18.9 μmol/L	1.2 nmol/L	[89]
	Cu-TCPP(Fe)	*S. aureus*	POD	10–7.5 × 10^7^ CFU/mL	6 CFU/mL	[90]
	MIL-88@Pt@MIL-88	MicroRNA-21	POD	1 fmol/L–1 nmol/L	0.29 fmol/L	[91]
SERS	AuNPs@MIL-101	Glucose Lactate	POD	10–200 μmol/L 10–200 μmol/L	4.2 μmol/L 5.0 μmol/L	[92]
	AgNPs@MIL-101	Cholesterol	POD	20 nmol/L–50 μmol/L	10.49 nmol/L	[93]
	MIL-100(Fe)@AuNPs	Histamine	POD	5 × 10^−3^–10^−11^ mol/L	3.9 × 10^−12^ mol/L	[94]
	AuNPs/Cu-TCPP(Fe)	Glucose	POD	0.16–8 mmol/L	3.9 μmol/L	[95]

## Data Availability

Data are contained within the article.
